# Randomized Controlled Trials of Rehabilitation Services in the Post-acute Phase of Moderate and Severe Traumatic Brain Injury – A Systematic Review

**DOI:** 10.3389/fneur.2019.00557

**Published:** 2019-06-06

**Authors:** Cecilie Røe, Cathrine Tverdal, Emilie Isager Howe, Olli Tenovuo, Philippe Azouvi, Nada Andelic

**Affiliations:** ^1^Department of Physical Medicine and Rehabilitation, Oslo University Hospital Ulleval, Oslo, Norway; ^2^Faculty of Medicine, Institute of Clinical Medicine, University of Oslo, Oslo, Norway; ^3^Research Centre for Habilitation and Rehabilitation Models and Services, Faculty of Medicine, Institute of Health and Society, University of Oslo, Oslo, Norway; ^4^Department of Neurology, University of Turku, Turku, Finland; ^5^Turku University Hospital, Turku, Finland; ^6^Service de Médecine Physique et de Réadaptation, Hôpital Raymond-Poincaré, Garches, France; ^7^Université de Versailles Saint-Quentin, Montigny-Le-Bretonneux, France

**Keywords:** rehabilitation, services, traumatic brain injury, randomized trials, post-acute

## Abstract

**Background and aims:** There is a gap in knowledge regarding effective rehabilitation service delivery in the post-acute phase after traumatic brain injury (TBI). Recently, Gutenbrunner et al. proposed a classification system for health-related rehabilitation services (International Classification System for Service Organization in Health-related Rehabilitation, ICSO-R) that could be useful for contrasting and comparing rehabilitation services. The ICSO-R describes the dimensions of Provision (i.e., context of delivered services), Funding (i.e., sources of income and refunding), and Delivery (i.e., mode, structure and intensity) at the meso-level of services.

We aim to:

-Provide an overview of randomized, controlled trials (RCTs) with rehabilitation service relevance provided to patients with moderate and severe TBI in the post-acute phase using the ICSO-R as a framework; and

-Evaluate the extent to which the provision, funding and delivery dimensions of rehabilitation services were addressed and differed between the intervention arms in these studies.

**Materials and methods:** A systematic literature search was performed in OVID MEDLINE, EMBASE, CINHAL, PsychINFO, and CENTRAL, including multidisciplinary rehabilitation interventions with RCT designs and service relevance targeting moderate and severe TBI in the post-acute phase.

**Results:** 23 studies with 4,644 TBI patients were included. More than two-thirds of the studies were conducted in a hospital-based rehabilitation setting. The contrast in Context between the intervention arms often co-varied with Resources. The funding of the services was explicitly described in only one study. Aspects of the Delivery dimension were described in all of the studies, and the Mode of Production, Intensity, Aspects of Time and Peer Support were contrasted in the intervention arms in several of the studies. A wide variety of outcome measures were applied often covering Body function, as well as the Activities and Participation domains of the International Classification of Functioning, Disability, and Health (ICF).

**Conclusion:** Aspects of service organization and resources as well as delivery may clearly influence outcome of rehabilitation. Presently, lack of uniformity of data and collection methods, the heterogeneity of structures and processes of rehabilitation services, and a lack of common outcome measurements make comparisons between the studies difficult. Standardized descriptions of services by ICSO-R, offer the possibility to improve comparability in the future and thus enhance the relevance of rehabilitation studies.

## Background

Traumatic brain injury (TBI) is a worldwide public health problem and can result in long-term disability (1–3) with the need for extensive and highly specialized initial health care provision, followed by comprehensive rehabilitation efforts (4). Physical, cognitive and emotional problems, including inability to return to full- or part-time work, as well as diminished quality of life, are frequent long-term consequences of TBI. Effective delivery of rehabilitation services and integration of medical perspectives, as well as vocational, educational and community support, are deemed necessary to meet the complex needs of this population. Services derive from the act of serving and refer to the provision of intangible products offered to persons with health conditions. Rehabilitation services in particular imply strategies targeting subsequent disability (5). Service delivery can be viewed from societal, institutional and individual perspectives. These different levels are often referred to as macro-level, including policy and financial aspects, meso-level, including organization and availability of services, and micro-level, including accessibility and content of services provided to an individual patient (6). Donobedian (7) described the quality of services as a causal relationship among the attributes of setting, the process of care, and the outcome. Evaluating the quality of rehabilitation services is important at every level, but the complexity of services and hence the challenges of evaluation can increase when moving from the micro- to the meso- and macro-levels.

A wide variety of rehabilitation interventions have been developed and evaluated with respect to content and outcomes for different functional problems after TBI (8). Service delivery to patients with severe TBI has focused on the acute phase, underpinning the importance of early initiated and well-organized delivery (9–11). Less is known about effective rehabilitation service delivery in the postacute and later phases, at least for the general TBI population (12, 13) and reviews focusing on this issue are warranted (14). Furthermore, the structure and process of care are seldom described, although they clearly impact the outcomes of TBI (15). The lack of a framework for depicting differences in service delivery could contribute to the scarce knowledge regarding optimal rehabilitation service delivery. Recently, Gutenbrunner et al. (16) proposed a classification for rehabilitation services, the International Classification System for Service Organization in Health-related Rehabilitation (ICSO-R), describing the meso-level of heath care. The ICSO-R is based on three dimensions Service provision,” Funding and Delivery each of which has a more extensive list of categories and subcategories that characterize rehabilitation services (17). The classification builds on the conceptual framework by Meyer et al. (5) describing health-related rehabilitation services according to their organizational setting including technical and human resources in addition to their goals.

The classification was developed in order to cover the gap between classifications at the micro and macro level of health care exemplified by the International Classification of Functioning, Disability and Health (ICF) (18) and the International Classification of Health Accounts (ICHA) (19). The ICSO-R intended to provide tools for analyzing provision and delivery of rehabilitation services. Based on these assumptions the classification might also be useful for contrasting and comparing rehabilitation services across different care facilities at the local, regional and country levels. Thus far, the lack of framework has hampered a systematic approach to the service aspects in the existing rehabilitation literature and how these aspects influence outcomes (20). There is an urgent need for prognostic models in TBI facilitating comparative audits of services among hospitals, other health care settings and countries (21). The Service provider dimension of ICSO-R describes the framework of the institution, organization, the resources and quality assurance and could be applied to evaluate where, by whom and in which context the service is delivered. The Funding dimension describes the main sources of income and funding of the services (i.e., diagnosis-related groups, per-day payment or other forms of services refund.). Finally, the Delivery dimension contains the main strategies (i.e., preventive, curative, rehabilitation, supportive or other strategies) delivered to the users, aspects of intensity and duration of intervention and the way the service is organized, and can be used in order to evaluate what, for what and how the services are delivered (17). Hence, the ICSO-R may serve as a tool for such comparative audit.

Using the ICSO-R as a framework, the current review aims to provide an overview of randomized, controlled trials (RCTs) with rehabilitation service relevance provided to patients with moderate and severe TBI in the post-acute phase and to evaluate the extent to which organizing, funding and providing of rehabilitation services were addressed.

## Materials and Methods

A systematic literature search was conducted to identify controlled trials evaluating the effects of rehabilitation services or rehabilitation interventions with service implications.

## Inclusion Criteria and Definitions

Studies targeting adults (>17 years old) with moderate or severe TBI and providing rehabilitation following the acute phase were included. TBI was defined as “an alteration in brain function, or other evidence of brain pathology, caused by an external force” (22). “Following the acute phase” was defined as rehabilitation occurring after discharge from the trauma center/acute-care hospital. A multidisciplinary approach was defined as at least two professions involved directly in the delivery of the intervention. Service relevance was operationalized to differences in the delivery, funding, or provision of the services between the intervention arms in the studies. A librarian was consulted to elaborate a thorough search strategy. Potential articles of interest in the English language were identified through a systematic search of the Medline (OVID), EMBASE, Cumulative Index of Nursing and Allied Health Literature (CINAHL), PsycINFO, and Cochrane Central Register of Controlled Trials (CENTRAL) databases (November 2016) with updated searches for 2016 through July 3, 2018, revealing a total of 2,970 hits (Appendix 1). In order to retrieve the highest possible number of relevant articles two filters were applied: the Cochrane Highly Sensitive Search Strategy for identifying randomized trials in MEDLINE: sensitivity-maximizing version (2008 revision) (https://training.cochrane.org/handbook); and the best balance of sensitivity and specificity filter for Therapy. These strategies build on optimizing the thesaurus terms, and adapted to the individual databases as recommended http://handbook1.cochrane.org. http://hiru.mcmaster.ca/hiru/HIRU_Hedges_home.aspx#Hedges. These filters were combined with “OR” in the search strategy in order to increase the number of hits. The filters were applied to the Medline and EMBASE searchs, but not in the search in the other databases. Hence these filters are assumed to increase and not limit the relevant hits.

## Review Process

**Figure 1:**
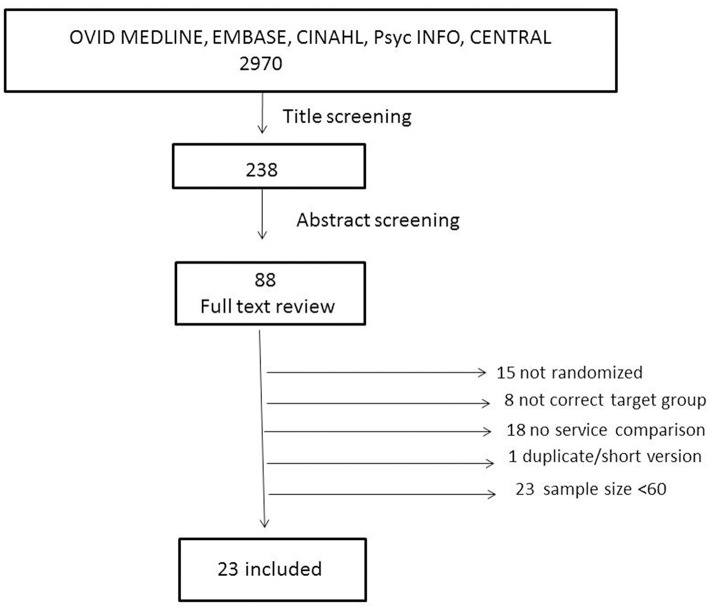
Flow chart of inclusion process.

The titles of the 2,970 studies were screened for eligibility, with supplementary evaluation of the abstracts when necessary (238 studies). A total of 88 studies were identified as candidates. These studies were evaluated independently by two of the authors regarding fulfillment of the inclusion criteria, and when in doubt, full-text manuscripts were assessed. Based on consensus 46 studies were found to meet the inclusion criteria. In 23 of the studies the sample size was lower than 60. Inter individual variations in functional outcomes vary across the specific outcome measures and so do the power estimates for needed number of patients. However, power around 80% and with detection of group differences around half the standard deviation is often recommended (23). Thus, studies with n<60 were excluded *post-hoc* in order to avoid studies with too low power. Hence, 23 studies were included in the final analyses (Figure 1).

## Quality Evaluation

The quality of the studies was evaluated by three researchers according to their adherence to the CONSORT guidelines (24) and was scored according to the Cochrane recommendations (25). Risk of bias in the included studies was assessed in 12 domains: adequate randomization method; concealed allocation; blinding of participants; blinding of care providers; blinding of outcome assessors; dropout rate described and <20% for short-term and 30% for long-term outcomes; intention to treat, i.e., all participants were analyzed according to their randomized group allocation; unselective reporting, i.e., results were provided from all prespecified outcomes; groups similar at baseline regarding demographics and other important clinical characteristics; similar or absent co-interventions; adequate compliance with the interventions; and similar timing of primary outcomes for the intervention and control groups. The score of 1 was given if the domain fulfilled the Cochrane recommendations and 0 if not. Hence, the total score had a possible range from 12 (highest level of quality) to 0 (lowest level of quality). Two pairs of raters evaluated the studies. Each rater conducted independent evaluations, and agreement between raters in each pair was provided. When in doubt, the domain was scored as 0. A consensus-based total score was subsequently elaborated. A data extraction sheet was elaborated capturing the type of randomization, number of involved study centers, sample size and age of the participants, as well as outcome measures.

## Data Analysis and Statistics

The data were summarized descriptively, and agreement between researchers was assessed by Pearson's correlation coefficient (r). The frequency of described dimensions and categories of service provision according to ICSO-R (15), with the predefined selection of descriptors suggested by Røe et al. (26), are reported. Service provision was categorized regarding Location (i.e., country), Organization (public or private), Context (hospital or community), Facility (rehabilitation or general medical) and Profit orientation (yes/no). The Funding dimension was assessed according to public, private or insurance-based sources of money. For the Delivery dimension Strategy of the intervention (diagnostic/therapeutic/management/prevention), Target group (TBI specific or not), Service goals (improvement of body function/activities and participation/adaptation to environmental factors), Team structure (interdisciplinary/multidisciplinary/single disciplines) and Mode of production, categorized as inpatient or outpatient delivery (inpatient or not), were assessed. We also attempted to disentangle whether these dimensions and categories varied between the intervention arms in the studies and summarized the main dimension/category differences. For the categories of Intensity, Aspects of time and Other, we could not apply the predefined terms from Røe et al. (26) we did not find applicable equivalents and used the descriptive approach by Kiekens et al. (17). Main outcome measurements from the selected studies are reported, along with the dimensions of the International Classification of Functioning, Disability and Health (ICF) (Body function, Activity and Participation, Environmental factors).

## Results

### Descriptions of Studies

A parallel group design was applied in 22 of the included studies. The studies were usually conducted within a single center (*n* = 20), and none of the studies included more than 3 sites. The median sample size was 120, ranging from 60 to 1,156 included subjects, with 4,644 participants altogether. The mean age was 39 years old in both the intervention and control groups, with the mean age ranging from 24 to 57 years old in the individual studies. Studies targeted the effects of different contents and intensities of rehabilitation across physical, emotional, cognitive and vocational strategies, as well as comparing rehabilitation with waiting list and in- and outpatient services (Tables 1, 2). In some of the studies, there was overlap between either participants or interventions. The studies by Winter et al. (39) and Moriarty et al. (40) represented the same intervention, focusing on the effects on the patients and their families, respectively. In the two studies by Bell et al., the intervention was conducted in a single center (28) and subsequently evaluated in a multicenter study (29) The studies by Wade et al. illustrated a replicated intervention (47, 48).

**Table 1:** Studies with differences between the intervention arms within the “Provider” dimension. Main outcomes as reported by the authors and the ICF dimensions covered.

**Included studies**	**Content intervention**	**Content control**	**Main outcome (ICF dimensions covered by all outcome measures)**
**DIFFERENCES IN RESOURCES AND CONTEXT**			
^*^Bedard et al. (27)	10 weeks of mindfulness- based cognitive therapy	Waiting list	*Symptoms of depression using the Beck Depression Inventory-II* (Body function)
^*^Bell et al. (28)	Telephone-based motivational interview post-discharge	Standard follow-up groups	*Composite outcome* (FIM, DRS, CIQ, FSE, GOS-E, EuroQol, NFI, PQOL, SF-36 and BSI) (Body function, Activities and Participation)
Bell et al. (29)	Scheduled telephone intervention	Treatment as usual	*Composite outcome* (FIM, DRS, GOS-E, Part-O) (Body function, activities and participation)
Berry et al. (30)	Individualized problem- solving intervention provided to family caregivers	Education-only control group	Caregivers: *Social Problem Solving Inventory-Revised, the Center for Epidemiological Studies-Depression scale the Satisfaction with Life scale, and a measure of health complaints* Patients: *Hamilton Depression Scale* (Body function)
^*^Bombardier et al. (31)	7 scheduled telephone calls, information, problem solving behavioral activation sessions over 9 months	Treatment as usual	*Brief Symptom Inventory-Depression (BSI-D) subscale, Neurobehavioral Functioning Inventory-Depression subscale, and Mental Health Index-5* (Body function)
Brenner et al. (32)	Health and wellness therapy	Waiting list	*Health Promoting Lifestyle Profile-II* (Activities and Participation)
^*^Cicerone et al. (33)	Intensive cognitive rehabilitation (15 h/w over 16 w)	Standard neurorehabilitation with individual, discipline-specific therapies	*Community Integration Questionnaire (CIQ) and Perceived Quality of Life scale (PQOL)* (Activities and Participation)
Heskestad et al. (34)	Cognitive-oriented consultation two weeks after the injury	No intervention	*Main outcome not stated but Postconcussion symptoms, Beck Depression Inventory, Epworth Sleepiness scale, Fatigue Severity Scale and SF-36 reported* (Body function, Activities and Participation)
Hoffman et al. (35)	Structured aerobic exercise regimen for 10 weeks	No treatment	*Beck Depression Inventory* (Body function)
McMillan et al. (36)	Group 1. Attention control training for 5 sessions over 4 weeks Group 2. Exercises	Control	*Self-report measures of cognitive function, mood or symptom reporting* (Body function, Activities and Participation)
^*^Ponsford et al. (37)	Group 1. Adapted cognitive behavioral therapy (CBT) Group 2. Non-individualized CBT	Waiting list	*Hospital Anxiety and Depression Scale (anxiety subscale), Depression, Anxiety and Stress Scale (depression subscale)* (Body function, Activities and Participation)
Sander et al. (38)	Brief intervention for modifying alcohol expectancies	Standard care	*Alcohol Expectancy Questionnaire-III Global Positive Expectancies and Cognitive and Physical Impairment scales; Readiness to Change Questionnaire; problem alcohol use* (Activities and Participation)
^*^Winter et al. (39)^*^Moriarty et al. (40)	Community re-integration focused on home based rehabilitation with home visits	Standard outpatient clinical care	*Target outcomes reflecting veterans' self- identified problems and self-rated functional competence (W) Family member depressive symptomatology, caregiver burden, caregiver satisfaction, acceptability of the intervention (M)* (Body function, Activities, Participation, Environmental factors)

* *Indicates a statistically significant difference between the intervention arms in one or more of the study outcomes*.

**Table 2:** Studies with differences between the intervention arms in the Delivery dimension. Main outcomes as reported by the authors and the ICF dimensions covered.

**Included studies**	**Content intervention**	**Content control**	**Main outcome (ICF dimensions covered by the outcome measures)**
**DIFFERENCES IN TEAM STRUCTURE**			
^*^Rath et al. (41)	Problem solving-focused group treatment	Conventional neuropsychological rehabilitation	*Not stated but Cognitive skills, Psychosocial function and Problem solving assessed* (Body function, Activities and Participation)
**DIFFERENCES IN MODE OF PRODUCTION**			
Bowen et al. (42)	Pre-discharge Interdisciplinary rehabilitation	1.Post-discharge interdisciplinary rehabilitation2.Outpatient treatment as usual	*The Wimbledon Self-Reported Scale of Emotions, Katz Adjustment Scale for Social Behavior, Cognition (Logical Memory and Wechsler Memory Scale Revised)* (Body function, Activities and Participation)
Salazar et al. (43)	Intensive, 8-week, in-hospital cognitive rehabilitation program	Home rehabilitation program with weekly telephone support	*Return to gainful employment and fitness for military duty* (Activities and participation)
^*^Vanderploeg et al. (44)	Cognitive didacticism with integrated interdisciplinary inpatient rehabilitation	Functional-experiential with integrated interdisciplinary rehabilitation	*Functional independence in living and return to work and/or school* (Activities and participation)
**DIFFERENCES IN INTENSITY**			
^*^Powell et al. (45)	Outreach treatment for two sessions per week for a mean of 27 weeks in a community settings	*Information with one home visit*	*Barthel index, the Brain Injury Community Rehabilitation Outcome-39* (Activities and Participation)
Slade et al. (46)	67% increase in intensity of inpatient therapy	Usual inpatient therapy	*Length of hospital stay*
**DIFFERENCES IN ASPECTS OF TIME**			
Wade et al. (47)^*^Wade et al. (48)	Early intervention (telephone or face-to-face counseling)	Usual follow-up	*Rivermead head injury follow-up questionnaire (97 + 98)*, Rivermead Postconcussion* Symptoms Questionnaire (97)* (Body Functions, Activities, and Participation)
**DIFFERENCES IN PEER INVOLVEMENT**			
^*^Hanks et al. (49)	Mentor treatment after discharge	Treatment as usual	*Peer mentoring questionnaire; brief Symptom inventory-18, family assessment*
			*Device, Coping Inventory for Stressful Situations; Short Michigan Alcohol Screening Test, Medical Outcomes Study 12- Item Short-Form Health Survey, Community Integration Measure* (Body function, Activities and Participation, Environmental Factors)

* *Indicates a statistically significant difference between the intervention arms in one or more of the study outcomes*.

### Quality of Studies

The quality of the studies was rated 7.00 (SD 2.11) and 6.61 (SD 2.02) by the two raters, with a high correlation between the raters (*r* = 0.94). At the single item level, the assessors had different scores on 5.43% (15) of the items, (concealment 3, co-intervention 8, compliance 4). See Appendix 2 for the consensus based quality ratings for each study and items. The most common cause of a reduced quality score was lack of blinding, with only one study designed to allow for blinding of patients and none obtaining blinding of care providers. In addition, concealment of group allocation was poorly described in some of the studies, and the evaluation of absent or similar co-interventions across intervention arms was challenging.

### Targeted Meso-Level Aspects of Services

The studies emerged from five different countries. A total of 14 of the studies were from the US, followed by 6 from the UK and one from each of the countries Canada, Australia and Norway. The organization was defined as public in 3 of the studies and not clearly stated in the remainder.

In one of the studies, the context was not possible to identify for the control group. In 70% of the studies, one or more of the interventions were conducted in hospitals, and in 30% of the studies, the interventions were conducted in the communities. In the studies conducted in hospital setting one or more of the intervention arms were performed in rehabilitation units and only one study included interventions confined only to a general hospital unit. None of the studies were conducted in nursing homes. In general, the studies provided no explicit information about the profit orientation of the services. In line with this finding, the funding dimension was impossible to determine in all except for one study (42).

Within the delivery dimension, statements specifically addressing service delivery according to the categories in ICSO-R were lacking. However, based on the information in the studies we found that 74% of the studies included a therapeutic strategy for the interventions, 17% were primarily managing, and 9% preventing in nature. In total 78% of the studies included improvement in body function as a service goal, and one third of the studies included multiple goals for the services. Only 22% of the studies included environmental factors as a goal for the interventions. In 4% of the active interventions and 52% of the control interventions, the team structure providing the services was difficult to disentangle. Only 13% of the active, interventions and 8% of the control interventions were deemed to be interdisciplinary, whereas 39% of the active and 17% of control interventions were multidisciplinary interventions. In more than 85% of the studies, the services were outpatient based.

### Outcomes

The outcome areas covered were physical, cognitive and mental, and neuropsychological assessment of cognitive functions, as well as activity, and participation components and composite scores covering global functioning were also applied (Tables 1, 2). Although, a wide variety of outcome measurements were applied with sparse overlap between studies all studies except Slade et al. (46) included functional outcome covering one or more of the ICF dimensions. The environmental factor was covered generally only through caregiver burden outcomes. In addition to the ICF dimensions, well-being and satisfaction, as well as quality of life, were addressed in the outcomes. In total, 12 studies reported statistically significant differences between the intervention and control groups in one or more of the outcomes (Tables 1, 2). All studies addressed symptom burden or functional problems.

### Intervention arm Contrasts Regarding Rehabilitation Services

Within the Provider dimension we did not find any studies in which Location, Organization (Public/Private) or Profit orientation varied between the intervention arms. In 14 of the studies we identified differences regarding Context and Resources (Table 1). These studies typically compared interventions with a waiting list or “treatment as usual” condition. Usually both Context and Resources varied between the intervention arms. The main impression is that additional rehabilitation Resources, as well as Context, influenced the outcomes in these studies, with reported effects on one or more of the outcomes in 7 of the studies. Human resources varied between the interventions in most of the studies (Table 1). In several of the studies, there were additional differences in Delivery aspects as well.

We choose to define Mode of production as the main Service difference among the interventions in the study by Bowen et al. (42), but context differences also existed between the intervention arms. Total of 9 studies were classified with the main differences between the intervention arms in the categories of the Delivery dimension and with variations in Team structure, Aspects of time, Intensity and Peer involvement (Table 2). The Team structure variations were related to the group or more individually based service delivery (41). As expected, Target groups were kept constant across the intervention arms. Strategy was also unchanged across all of the intervention arms. In five of the studies, statistically significant outcome differences between the intervention arms were identified (Table 2).

## Discussion

The present review provides an overview of randomized, rehabilitation trials with service provision relevance in the post-acute phase after moderate and severe TBI. Half of the studies reported statistically significant differences between interventions in one or more of the outcome measurements. That most of the studies focused on the rehabilitation strategy i.e., content, with implicit, more than explicit, variations in the service provision and delivery, was a challenge. Furthermore, the lack of universal terminology and reporting standards for the service aspects, as well as the diversity of interventions and outcome measures, prohibited analysis of the effects of service provision across studies, as well as metaanalytic approaches.

Rehabilitation service provision is complex and varies across health care settings and countries, with a lack of synthesized information regarding effective organization of services based on randomized trials (50). Successful outcomes at the patient level are dependent on the organization, capacity and quality of rehabilitation services at the macro-, meso- and micro-levels (51). Several reviews have been conducted regarding the effects of different interventions and treatment modalities targeting physical, cognitive and emotional problems after TBI (8, 52–59). However, very few evaluated directly the effects of differences in service provision and delivery supporting the gap in knowledge regarding post-acute services for TBI at the meso-level. Service provision and delivery related factors may thus influence outcome across reported significance. The review also illustrated that, when applying a structured framework, differences in service provision and delivery could be deduced from intervention studies, primarily evaluating programs at the micro-level.

The implicit components of services included in the treatment and interventions compared in clinical studies are an enormous challenge regarding the evaluation of effective service models. Thus, synthesizing evidence regarding effective components in service provision is also difficult. ICSO-R provided a tool for systematizing important elements of services across intervention arms. Describing the studies according to ICSO-R did, however, indicate that important elements of services varied across intervention arms and could influence the outcomes. One might argue that the majority of the studies were conducted before the ICSO-R was published. However, the aspects of services addressed in the ICSO-R have been relevant to service provision for decades (7). Laver et al. (14) conducted a systematic review regarding evidence for organizing health care for people with acquired brain injury, identifying 8 studies of TBI. When excluding studies with mainly mild TBI and those conducted in the acute phase, the studies included in Lavers' review overlapped with the present review. However, a main limitation is the current lack of subcategories in the ICSO-R, overlapping categories, and a lack of definitions. We applied some predefined subcategories suggested by Røe et al. (26). These predefined categories clearly failed to capture the main differences between studies regarding the intensity and timing of the intervention, as well as regarding team structure. These subcategories were developed to reduce overlap between categories, which is inherent to the original ICSO-R. This adjustment might have biased our results with over reporting of context differences and underreporting of organizational and facility differences. The new version of the ICSO-R that is being elaborated might provide a better tool for analyzing the effects of service provision in the future. The present review also illustrated that improving aspects of better information regarding service provision and delivery could be gained from the existing literature.

We categorized the studies according to the main service dimension and categories differing between the intervention arms (Tables 1, 2). Based on this approach, we identified aspects of service provision and delivery that clearly could impact evaluations of the effects in these studies. Very few studies clearly stated whether the services were private or publicly organized. These aspects could impact patient selection beyond the socio-demographic characteristics reported in the studies. We did not identify any studies focusing on the funding dimension, although both public and private organization of services and type of funding are very important aspects for policy makers and stakeholders (60). Patients' payments and refunding of the services influence outcomes, but they were poorly described in the included studies. Future studies should address this aspect more directly because resources constitute a barrier to the implementation of services (61).

Neither the dimensions nor the categories in the ICSO-R are mutually independent. In the studies comparing specified interventions with waiting lists or usual care treatment (Table 1), the differences in the service provider dimension were evident, often with different contexts and more resources in the active interventions, compared to treatment as usual care and waiting lists. However, covariance with delivery aspects was inevitable in these studies. Some of the studies included in the present review rather explicitly targeted the mode of production (51–54), but comparing in- and outpatient services generally also implies differences in provision, i.e., context and facilities.

Covariance among categories within each dimension of the ICSO-R was even more evident. For example, in Bowen et al. (42), the mode of production with pre- and post-discharge comparisons of rehabilitation interventions co-varied with differences in the timing category (Table 2). In the studies with several intervention arms, the nature of differences could vary between the intervention arms, rendering the classification challenging. In the study by Bowen et al. (42), two of the intervention arms varied regarding the mode of production, whereas the third arm (treatment as usual) could be evaluated as having different service provision aspects (Context and Resources).

The ICSO-R was specifically developed to cover rehabilitation services at the meso-level (15). The primary goal of the majority of included studies in this review focused on the content of the interventions, i.e., the micro-level of services (5). The distinction between service delivery at the meso-level and content at the micro-level might not always be clear cut (22). Several of the studies identified in our literature search evaluated the effects of different neuropsychological approaches. Since intensity is a category in the ICSO-R, studies with slightly different intensities of rehabilitation were included, although intensity could be evaluated as an important aspect of the content. Difficulties in clear-cut distinctions between the meso- and micro-levels of services are accompanied by a lack of invariance across such aspects. Content of treatment is not included in the ICSO-R but is needed to assess the effects of rehabilitation. To address the effects of different service provision components on outcomes, a more specified, detailed and universally applied system for service provision and delivery is needed. To some extent, the needed process could be compared with the development of ICF (18). Hence, 17 years after the launching of ICF, its application as a framework for systematizing outcome evaluation is increasing (62). A revised version of the ICSO-R represents one step toward this goal. However, a universal and not too complicated taxonomy for the content of effective ingredients is also urgently needed (63).

Consequently, the effects of differences in rehabilitation services cannot be directly determined from the present review. Nevertheless, it is reasonable to assume that resources and contextual factors did contribute to the documented differences between the intervention arms. It was an important aspect of the aim to evaluate in-hospital vs. at-home services in several of the studies, i.e., differences in mode of production and context (42, 43). These studies failed to document major differences in outcomes, except for better patient satisfaction with at-home services (Table 2). This finding is in contrast to the experience of patients with stroke, in whom early supported discharge showed superior efficacy over in-hospital services (64). This difference might be caused by greater variability in the needs and goals of patients with TBI and also methodological limitations in the studies included in the present review. In contrast, Winter et al. and Moriarty et al. (25) documented improvements in individually targeted outcomes for patients, as well as their relatives in people centered in home care, compared to “treatment as usual.” However, in these studies, the intensity/amount of rehabilitation could also have been different and impacted the outcome (Table 2). Variable influence in outcomes was indicated by variations in team structure, intensity and aspects of time, while Hanks et al. (49) study supported improved outcomes by peer involvement in the interventions.

The present review also underpins that replication of interventions across service providers, and delivery aspects might be important. The study by Bell et al. (28) indicated positive results of a telephone follow-up in a single center study, but it was not replicated with a multicenter design (29). Underpinning the need for validation studies, Wade et al. (48) documented significant results in their replicated study with early intervention (aspects of time). All, except one of the included studies targeted symptoms or functional problems as outcome. Increased use of common data elements and linking approaches between measurements may facilitate better comparison between studies in the future (65, 66).

The quality of the included studies varied, and reaching a maximum score might not be possible due to the lack of possibility of blinding patients and rehabilitation providers. We applied the CONSORT guidelines and scored the quality according to Furlan et al. (25) Rehabilitation might require an adapted scoring system acknowledging the special challenges in this field (67). For example, although not blinded to the intervention itself, blinding to its aims and mechanisms could be the best possible choice and could be acknowledged. The quality evaluation clearly revealed that improvement is needed in describing the concealment of randomization. Possibly more important for the results and their interpretation are better assessment and description of co-interventions. To evaluate the components related to the differences and effects in randomized rehabilitation trials, improved description of the provision and delivery of the services, along with the content of the interventions, is needed and should be included in quality evaluation systems.

The main limitations of the present review are the lack of inclusion and exclusion criteria for the ICSO-R categories and the lack of common descriptors of the services in the studies. This is an obstacle for the inclusion process of studies as well as for analyzing and reporting the influence on outcome in the studies.

## Conclusion and Clinical Relevance

A lack of uniformity of data and collection methods, the heterogeneity of structures and processes of rehabilitation services, and the lack of common outcome measurements made the study results less generalizable and the comparison between studies difficult. Standardized descriptions of services, including provider, funding and delivery dimensions, could improve the service relevance of rehabilitation studies and give valuable information to many different stakeholders. A shorter version of ICSO-R with value sets may be needed for inclusion in rehabilitation studies description.

## Author Contributions

CR, CT, EH, OT, PA, and NA contributed to the theoretical background, framework for analysis and writing process. CR, CT, and EH conducted the data analysis and, together with NA, elaborated the first manuscript draft.

### Conflict of Interest Statement

The authors declare that the research was conducted in the absence of any commercial or financial relationships that could be construed as a potential conflict of interest.
