# Supplementary material for: Randomized Controlled Trials of Rehabilitation Services in the Post-acute Phase of Moderate and Severe Traumatic Brain Injury – A Systematic Review
Source: Front Neurol. 2019 Jun 6;10:557. doi: 10.3389/fneur.2019.00557 (PMC6563754; doi:10.3389/fneur.2019.00557)
Supplement: Supplementary file 1 [file Table_1.pdf]

Appendix 1.

Database: Ovid MEDLINE(R) Epub Ahead of Print, In-Process & Other Non-Indexed Citations, Ovid MEDLINE(R) Daily and Ovid MEDLINE(R) <1946 to Present>

----

1 craniocerebral trauma/ or brain hemorrhage, traumatic/ or brain stem hemorrhage, traumatic/ or cerebral hemorrhage, traumatic/ or head injuries, penetrating/ or intracranial hemorrhage, traumatic/ or subarachnoid hemorrhage, traumatic/ or skull fractures/

2 Brain Injuries/ and (trauma or traumas or traumatic* or fracture*).tw,kf.

3 ((head or heads or brain or brains or cerebral or cerebellum or craniocerebral or skull) adj3 (trauma or traumas or traumatic* or fracture*)).tw,kf.

4 or/1-3

5 Rehabilitation/ or (rehabilitation or rehabilitative or neurorehabilit*).tw,kf.

6 ((structur* adj3 care) or (process* adj3 care)).tw,kf.

7 (trauma care or tbi care or neurotrauma care or postacute care or post acute care or patient care or health care or (contin* adj2 care) or (discont* adj2 care)).tw,kf.

8 health services/ or community health services/ or home care services/

9 health services administration/ or "organization and administration"/

10 ((health adj3 service*) or (care adj3 transition*) or (care adj2 pathway*) or (clinical adj2 pathway*) or critical pathway*).tw,kf.

11 "continuity of patient care"/ or patient discharge/ or patient transfer/ or transitional care/

12 ((patient* adj3 transfer*) or (patient* adj3 transition*) or patient discharge).tw,kf.

13 patient care management/ or comprehensive health care/ or patient care planning/ or exp primary health care/ or progressive patient care/ or critical pathways/ or "delivery of health care"/ or exp "delivery of health care, integrated"/ or patient care team/

14 Health Facilities/ or Rehabilitation Centers/

15 ((health adj3 facilit*) or (health adj3 service*) or nursing home* or home based or (home adj3 care) or hospital or hospitals or (home adj3 setting*) or communit*).tw,kf.

16 organization & administration.fs.

17 or/5-16

18 4 and 17

19 (child* or pediatric or paediatric or infant*).ti.

20 (mild traumatic brain or concussion*).ti.

21 19 or 20

22 18 not 21

23 randomized controlled trial.pt.

24 controlled clinical trial.pt.

25 randomized.ab.

26 placebo.ab.

27 clinical trials as topic.sh.

28 randomly.ab.

29 trial.ti.

30 or/23-29

31 exp animals/ not humans.sh.

32 30 not 31

33 22 and 32
34 limit 22 to "therapy (best balance of sensitivity and specificity)"
35 33 or 34
36 limit 35 to english language
