# Supplementary material for: Randomized Controlled Trials of Rehabilitation Services in the Post-acute Phase of Moderate and Severe Traumatic Brain Injury – A Systematic Review
Source: Front Neurol. 2019 Jun 6;10:557. doi: 10.3389/fneur.2019.00557 (PMC6563754; doi:10.3389/fneur.2019.00557)
Supplement: Supplementary file 2 [file Table_2.pdf]

Appendix 2. Risk of bias as assessed according to the Cochrane recommendations.

Study	Randomization		Allocation		Blinding patients		Blinding provider		Blinding assessor		Drop out		Intention to treat		Selective outcome		Baseline		Co-intervention		Compliance		Timing Outcome	
	R1	R2	R1	R2	R1	R2	R1	R2	R1	R2	R1	R2	R1	R2	R1	R2	R1	R2	R1	R2	R1	R2	R1	R2
Bedard M (2014)(56)	1	1	0	0	0	0	0	0	1	1	0	0	0	0	1	1	1	1	0	1	0	0	1	1
Bell KR (2005)(29)	1	1	0	0	0	0	0	0	1	1	1	1	1	1	1	1	1	1	0	0	1	1	1	1
Bell KR (2011)(30)	1	1	0	0	0	0	0	0	1	1	1	1	1	1	1	1	0	0	0	0	1	0	1	1
Berry JW (2012)(57)	1	1	0	0	0	0	0	0	1	1	0	0	1	1	1	1	1	1	1	1	1		1	1
Bombardier CH (2009)(58)	1	1	0	0	0	0	0	0	1	1	1	1	1	1	1	1	0	0	0	0	1	0	1	1
Brenner LA (2012)(59)	1	1	0	0	0	0	0	0	0	0	1	1	1	1	1	1	1	1	0	0	1	0	1	1
Cicerone KD (2008)(60)	1	1	1	1	1	1	0	0	1	1	1	1	1	1	1	1	1	1	1	0	1	1	1	1
Heskestad B (2010)(61)	1	1	0	0	0	0	0	0	0	0	0	0	1	1	1	1	1	1	1	0	1	1	1	1
Hoffman JM (2010)(62)	1	1	0	0	0	0	0	0	1	1	1	1	1	1	1	1	0	0	0	0	0	0	1	1
McMillan T (2002)(63)	1	1	0	1	0	0	0	0	1	1	1	1	1	1	1	1	1	1	1	1	1	1	1	1
Ponsford J (2016)(64)	1	1	0	0	0	0	0	0	1	1	0	0	1	1	1	1	1	1	1	0	1		1	1
Sander AM (2012)(65)	1	1	0	0	0	0	0	0	1	1	0	0	1	1	1	1	1	1	1	1	0	0	1	1
Winter L (2016)(27)	1	1	0	0	0	0	0	0	1	1	1	1	1	1	1	1	0	0	0	0	0	0	1	1
Moriarty H (2016)(28)	1	1	0	0	0	0	0	0	1	1	1	1	1	1	1	1	1	1	1	1	1	0	1	1
Rath JF (2003)(35)	1	1	0	0	0	0	0	0	0	0	0	0	1	1	1	1	0	0	0	0	1	1	0	0
Bowen A (1999)(33)	0	0	0	0	0	0	0	0	0	0	0	0	1	1	0	0	1	1	0	0	0	0	1	1
Salazar RM (2000)(50)	1	1	0	0	0	0	0	0	0	0	1	1	1	1	1	1	0	0	0	0	1	1	1	1
Vanderploeg R (2008)(66)	1	1	0	0	0	0	0	0	1	1	1	1	1	1	1	1	1	1	1	0	0	1	1	1

Powell J (2002)(67)	1	1	0	0	0	0	1	1	1	1	1	1	1	1	1	1	1	1	0	0	1	1	1	1
Slade A (2002)(34)	1	1	0	0	0	0	1	1	1	1	1	1	1	1	1	1	1	1	1	1	1	1	1	1
Wade DT (1997)(31)	1	1	1	0	0	0	0	0	1	1	0	0	1	1	1	1	0	0	0	0	0	0	1	1
Wade DT (1998) (32)	1	1	0	0	0	0	0	0	1	1	0	0	1	1	0	0	1	1	1	0	0	0	1	1
Hanks RA (2012)(52)	1	1	0	0	0	0	0	0	0	0	0	0	1	1	0	0	1	1	1	0	0	0	1	1

Criteria assessed as not fulfilled are indicated 0, fulfilled 1. Rater one (R1), Rater two (R2)
